# Bacteria within the Gastrointestinal Tract Microbiota Correlated with Improved Growth and Feed Conversion: Challenges Presented for the Identification of Performance Enhancing Probiotic Bacteria

**DOI:** 10.3389/fmicb.2016.00187

**Published:** 2016-02-19

**Authors:** Dragana Stanley, Robert J. Hughes, Mark S. Geier, Robert J. Moore

**Affiliations:** ^1^Institute for Future Farming Systems, Central Queensland UniversityRockhampton, QLD, Australia; ^2^Poultry Cooperative Research Centre, University of New England, ArmidaleNSW, Australia; ^3^Pig and Poultry Production Institute, South Australian Research and Development Institute, RoseworthySA, Australia; ^4^School of Animal and Veterinary Sciences, The University of Adelaide, RoseworthySA, Australia; ^5^Research and Innovation Services, University of South Australia, Mawson LakesSA, Australia; ^6^Australian Animal Health Laboratory, CSIRO, GeelongVIC, Australia; ^7^School of Science, Royal Melbourne Institute of Technology University, BundooraVIC, Australia; ^8^Department of Microbiology, Monash University, ClaytonVIC, Australia

**Keywords:** energy assimilation, weight gain, caecum, gastrointestinal tract, feed conversion, digestive efficiency, microbiota, probiotic

## Abstract

Identification of bacteria associated with desirable productivity outcomes in animals may offer a direct approach to the identification of probiotic bacteria for use in animal production. We performed three controlled chicken trials (*n* = 96) to investigate caecal microbiota differences between the best and poorest performing birds using four performance measures; feed conversion ratio (FCR), utilization of energy from the feed measured as apparent metabolisable energy, gain rate (GR), and amount of feed eaten (FE). The shifts in microbiota composition associated with the performance measures were very different between the three trials. Analysis of the caecal microbiota revealed that the high and low FCR birds had significant differences in the abundance of some bacteria as demonstrated by shifts in microbiota alpha and beta diversity. Trials 1 and 2 showed significant overall community shifts, however, the microbial changes driving the difference between good and poor performers were very different. *Lachnospiraceae, Ruminococcaceae*, and *Erysipelotrichaceae* families and genera *Ruminococcus, Faecalibacterium* and multiple lineages of genus *Clostridium* (from families *Lachnospiraceae, Ruminococcaceae*, and *Erysipelotrichaceae*) were highly abundant in good FCR birds in Trial 1. Different microbiota was associated with FCR in Trial 2; *Catabacteriaceae* and unknown *Clostridiales* family members were increased in good FCR and genera *Clostridium* (from family *Clostridiaceae*) and *Lactobacillus* were associated with poor FCR. Trial 3 had only mild microbiota differences associated with all four performance measures. Overall, the genus *Lactobacillus* was correlated with feed intake which resulted in poor FCR performance. The genus *Faecalibacterium* correlated with improved FCR, increased GR and reduced FE. There was overlap in phylotypes correlated with improved FCR and GR, while different microbial cohorts appeared to be correlated with FE. Even under controlled conditions different cohorts of birds developed distinctly different microbiotas. Within the different trial groups the abundance of certain bacterial groups correlated with productivity outcomes. However, with different underlying microbiotas there were different bacteria correlated with performance. The challenge will be to identify probiotic bacteria that can reliably deliver favorable outcomes from diverse microbiotas.

## Introduction

The gastrointestinal tract (GIT) microbiota of humans and animals consists of complex populations that include many different species of bacteria along with smaller numbers of fungi, protozoa, and archaea (Zoetendal et al., 2006). Over the last decade our understanding of the diverse functional roles and interactions of the GIT microbiota has rapidly advanced (Zoetendal et al., 2006). For example, in a normally functioning microbiota-host interaction the microbiota expands the hosts biochemical potential, assists in the digestion of food, produces micronutrients, modulates the immune system, and interacts with the central nervous system (Gill et al., 2006; Turnbaugh et al., 2007; Mayer et al., 2015). A dysfunctional microbiota can induce metabolic, autoimmune and inflammatory diseases, and can seriously undermine gut function (Ferreira et al., 2014; Joyce and Gahan, 2014; Silva et al., 2015).

Understanding the role of GIT microbiota in determining the efficiency with which food is utilized and converted to body mass is of importance in both human health and production animal science. In human studies there is an emphasis on understanding the role that GIT microbiota may have in reducing obesity and promoting leanness whereas in production animal studies a major goal is to define the microbiota that most efficiently converts food to body mass; in particular muscle (Stanley et al., 2014). Fecal transfer experiments in mouse models of obesity have demonstrated that there are causal links between GIT microbiota composition and obesity (Turnbaugh et al., 2006) and studies of humans have demonstrated a correlation between GIT microbiota composition and obesity (Ley et al., 2006; Turnbaugh et al., 2006). Similarly, correlations between GIT microbiota composition and efficiency of energy extraction have been noted in chickens (Torok et al., 2011; Stanley et al., 2012, 2013a).

One of the goals of research aimed at defining the microbiota associated with high or low efficiency of energy retention from food is to develop ways to manipulate microbiota composition; either indirectly by modification and supplementation of food intake, or directly, for example, by the use of probiotic strains of bacteria. The current generation of probiotics have generally been indirectly identified by *in vitro* screening of strain collections to identify isolates with properties that are hypothesized to be indicative of *in vivo* performance; for example, resistance to bile acids and adherence to epithelial cells. However, there is little direct evidence that *in vitro* screening is a valuable approach (Morelli, 2000). Some current probiotics are not derived from the host in which they are intended to be used. Given the complexity and specificity of many natural host-microbe interactions it is perhaps not surprising that most probiotics, when not derived from the target host, need to be continually dosed. Even with continuous dosing the effectiveness of probiotics can be variable and unreliable (Ajuwon, 2015). One reason for such variability in performance may be the very varied microbiotas that are present in both humans and animals (Li et al., 2013; Stanley et al., 2013b). A probiotic may function effectively in one microbiota but not in another different microbiota.

The research described here takes an alternative approach in the first steps toward identifying bacteria that may have potential probiotic applications. Rather than start with a collection of bacterial isolates and complete a long series of *in vitro* and *in vivo* tests to determine if they can modify a particular host function we have started with birds which exhibit the desired host properties, i.e., efficient use of feed, and have analyzed the GIT microbiota to determine whether there are particular bacterial species that tend to be associated and hence may represent targets for isolation and use as probiotics to induce the desired property. Previous work has demonstrated that in chickens there are certain bacteria that are associated with a high ability to capture energy from food (Torok et al., 2008; Stanley et al., 2013a). Other work has shown a high degree of variation in the population structure of GIT microbiota between different flocks of birds (Stanley et al., 2013b). Therefore, the current experiments aim to determine if the bacterial species that are associated with improved food use are consistent across flocks with different underlying microbiotas or whether each flock of birds exhibits a distinct spectrum of bacteria associated with the desirable trait.

The goal of the research presented here is to determine if there are types of bacteria that are consistently associated with the efficient extraction and use of the energy content of food. We have taken the data from three animal trials that have previously been used to investigate inter- and intra-trial variability in GIT microbiota composition (Stanley et al., 2013b) and the relationship between caecal and fecal microbiota composition (Stanley et al., 2015) to now explore the relationship between GIT microbiota composition and a number of standard measurements of animal performance. Bacterial types that correlate with high performance represent good targets for future development as probiotics.

## Materials and Methods

### Animal Trials

Three animal trials were performed following the same procedures over a 5 months period. The animal trials (each with *n* = 96) were performed as previously described (Stanley et al., 2013b). Briefly, one-day old male Cobb 500 broiler chickens from the Baiada Hatchery, Willaston, SA, Australia, were transferred to a chick rearing pen in a fully environmentally controlled experimental animal facility. Feed was supplied *ad libitum* throughout the experiment and was comprised of 44.4% wheat, 17% soybean meal, 15% barley, 10% canola meal, 5% peas, 3.2% meat meal, 3% tallow, 1% limestone, 0.5% vitamin mix, and traces of salt, lysine HCl, DL-methionine and threonine. The three trials were identical in source of birds (same hatchery and breeding stock), animal housing used, environmental control, feed formulation and all other variables controlled. Moreover, the same batch of commercially prepared feed crumbles was used in all three trials and was stored under controlled cool and dry conditions for the duration of the trials. All three trials were conducted within a 5 months period. All birds within each trial were housed together for the first 13 days of life to ensure microbiota exchange through typical bird behavior, including coprophagy.

On day 13 chicks were transferred in pairs to 48 metabolism cages in a temperature controlled room (23–25°C). Initial placing in metabolic cages in pairs was done to minimize stress and allow the birds to adjust to cages. At day 15, birds were moved into individual cages. Individual caging allowed the precise assessment of individual feed intake, energy in feed, and unused energy remaining in feces. The experimental design eliminated competition for feed and reduced behavioral issues affecting feed intake. Single bird caging and individual measurements and sampling were implemented in order to allow direct correlation of microbiota structure and productivity measurements on a bird by bird basis. Birds were euthanized and necropsied on day 25 and caecal contents and cloacal swabs were collected from each bird. Samples from all birds from all three trials were sequenced.

Feed conversion ratio (FCR) was calculated as a ratio of feed eaten (FE) and weight gained. Thus birds with low FCR, that need less feed per kg gained, are the most efficient in converting feed to mass. Gross energy (GE) was measured in feed and in feces of each individual bird using a Parr isoperibol bomb calorimeter (Parr Instrument Company, Moline, IL, USA). Apparent metabolisable energy (AME) in MJ/kg dry matter, was calculated as {AMEdiet = [(GEdiet × FE) × (GEexcreta × dry excreta)]/FE/dry diet content}. Gain rate (GR) was calculated as [weight gain (g)/start weight (g)] and FC was total amount of FE during the 10 days measurement time period. All of the above measurements were taken from day 15 to day 25, during the time when single birds were housed in metabolic cages.

### Ethics Statement

The three animal trials were approved and monitored by the Animal Ethics Committees of the University of Adelaide (Approval No.S-2010-080) and the Department of Primary Industries and Resources, South Australia (Approval No. 08/10). All animal work was conducted in agreement with the national and international guidelines for animal welfare.

### DNA Preparation, PCR Amplification of 16S rRNA Gene Sequences, and Bioinformatic Analysis

DNA was prepared as detailed by Stanley et al. (2013b). Briefly, DNA was isolated using the method of Yu and Morrison (2004) and the V1-V3 region of the 16S rRNA gene was amplified (forward primer (Lane, 1991), 5′ AGAGTTTGATCCTGG 3′; reverse primer W31 (Snell-Castro et al., 2005), 5′ TTACCGCGGCTGCT 3′). Pyrosequencing was performed using a Roche/454 FLX+ instrument and Titanium chemistry according to the manufacturer’s instructions. Sff file processing was done using PyroBayes (Quinlan et al., 2008) and inspected for chimeric sequences with Pintail (Ashelford et al., 2005) and errors using Acacia (Bragg et al., 2012). Further trimming was done in QIIME v1.8 (Caporaso et al., 2010) with sequence length 300–600 bases, no ambiguous sequences, minimum average quality score of 30 and maximum of six bases in homopolymer runs. OTU picking was done using Uclust (Edgar, 2010). Taxonomy was assigned using Blast against the GreenGenes database (DeSantis et al., 2006) and QIIME v1.8 defaults. All samples represented by less than 1000 sequences were removed from the analysis. Rare OTUs with relative abundance less than 0.01% were removed from further consideration. Remaining analysis was done in QIIME and some data visualized in Megan (Huson et al., 2007) and Calypso (http://bioinfo.qimr.edu.au/calypso/).

Fecal samples (via cloacal swab) were also collected for all birds and analyzed identically to the caecal data. Due to the volume of information fecal data is not presented in this manuscript. Statistical comparisons between the 12 highest and 12 lowest performing birds were assessed using a QIIME-based *t*-test, while all birds in each trial are used in Person correlation analysis for each family, genus, and OTU in the dataset against all four variables. Alpha diversity comparisons were calculated using a two-sample non-parametric *t*-test and 10^6^ Monte Carlo permutations. Beta diversity was based on Adonis statistics and 10^6^ permutations. To identify phylotypes associated with high performance over a combination of performance variables we used a Random Forest machine-learning algorithm and RapidMiner software.

Complete sequencing data, including both fecal and caecal samples, is available from MG-RAST under project ID 4667472.3.

## Results

### Performance of the Three Flocks

**FIGURE 1:**
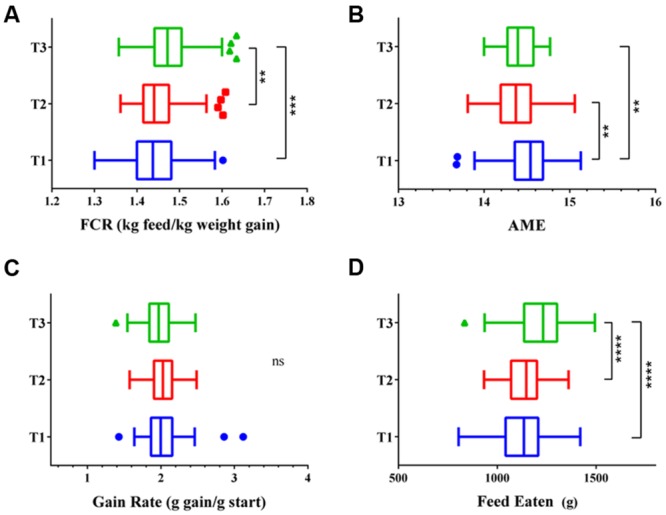
**Performance of the three flocks shown as (A) feed conversion ratio (FCR), (B) apparent metabolisable energy (AME), (C) gain rate (GR), and (D) feed eaten (FE).** Trial 1 is shown in blue, Trial 2 in red, and Trial 3 in green. ^∗^*p* < 0.01, ^∗∗^*p* < 0.005, ^∗∗∗^*p* < 0.001

**FIGURE 2:**
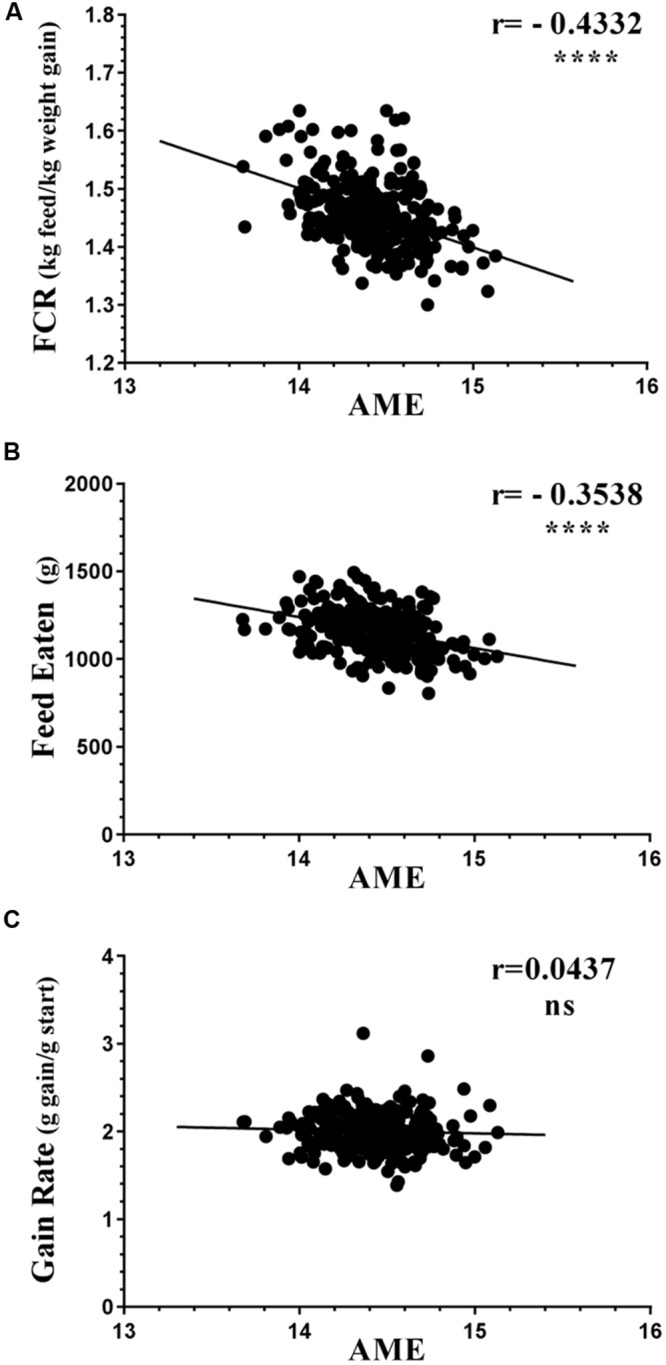
**Correlations of AME against (A) FCR, (B) FE, and (C) GR.** All birds from all three trials are represented as circles. The birds with higher AME values corresponding to better efficiency in energy extraction are likely to have lower (better) FCR, mostly due to eating less feed while having no change in gain rate (GR). Supplementary Figure S1 shows the same variables separately in each trial; demonstrating that this trend is reproducible.

All three flocks showed very good performance as indicated by FCR, AME, GR, and FE as performance measures (**Figure 1**). The four variables showed significant differences in all three trials between the 12 best and 12 poorest performing birds used in microbiota comparisons (Supplementary Figure S1). Comparison of performance measures across trials showed that there were no significant differences between trials in GR, however, there were significant differences in FE (*p* < 0.0001) with birds from Trial 3 consuming more feed than those from Trials 1 and 2. Birds from Trial 3 had the poorest FCR, lowest GR, and highest FE relative to the other two trials. However, all three trials, including Trial 3, performed above the breeder’s standard (Cobb-Vantress, 2013). Inspection of the correlations between the performance parameters identified AME as significantly negatively correlated with FCR and FE (**Figure 2**). Birds with higher AME values, corresponding to better efficiency in energy extraction, tend to have lower FCR due to lower feed consumption, while having no change in GR. This trend was observed in all three trials (Supplementary Figure S2).

### Overall Structure of the Caecal Microbiota from the Three Flocks

Comparison of the microbiota composition across the three replicate trials showed that there were highly significant differences between the trials; as high as at the phylum level (Supplementary Figure S3). There were substantial differences in the abundance of *Lactobacillus, Bacteroides, Ruminococcus*, and *Faecalibacterium* between trials. This inter-trial variability has been discussed previously (Stanley et al., 2013b); it was reported that 58% of all OTUs in the three trials were significantly (*p* < 0.05) differentially abundant between trials. It was also noted that Trial 3 had very different alpha diversity from the other two trials, having significantly more OTUs, especially rare microbiota (Stanley et al., 2013b). The differences between the trials warranted individual analysis of each trial to identify OTUs associated with productivity and then comparison of these differential OTUs across the trials. The caecal microbiota of the 12 best and 12 poorest performing birds from each trial, for each of the four variables, were analyzed to identify differential phylotypes. We used all samples from the trials to detect Pearson correlations between phylotypes at a family, genus and OTU level for FCR, AME, GR, and FE.

### Microbiota Correlated with Conversion of Feed to Body Weight

Although differences between the birds with highest and lowest variables (FCR, AME, GR, and FE) were significant (Non-parametric Mann–Whitney *U* test, (*p* < 0.0001) in all variables and all three trials (Supplementary Figure S1), FCR was the only performance variable that showed convincing difference in microbiota between good and poor performing birds. There were fewer microbiota differences between birds with good and poor AME, FE, and GR. The three trials showed very different microbiota correlations to FCR with Trials 1 and 2 responding strongly but differently, even at higher taxonomic levels, while the microbiota in Trial 3 birds showed comparatively mild responses to FCR extremes.

**FIGURE 3:**
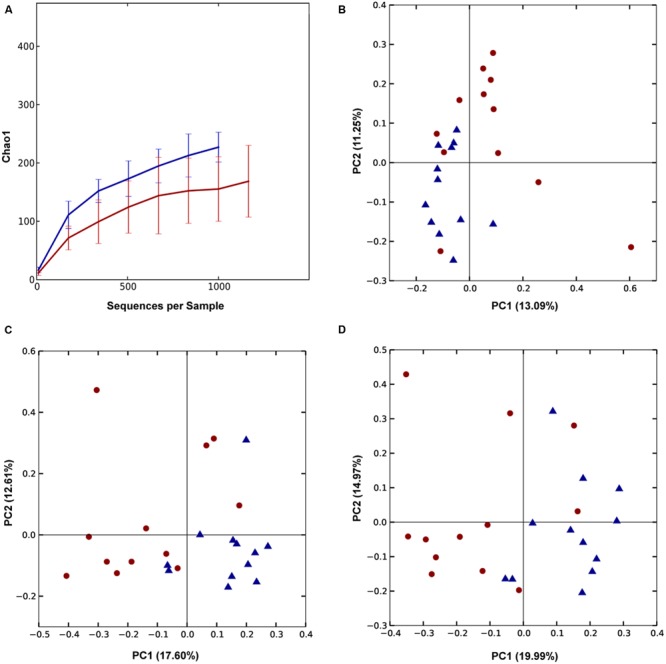
**Trial 1: Differences between high (red) and low (blue) FCR microbial communities. (A)** Alpha diversity metric Chao1 was significantly (*p* = 0.0021) higher in low FCR birds. **(B)** Unweighted UniFrac (*p* = 3E^-4^) PCoA plot; **(C,D)** PCoA plot of beta diversity using Canberra distance at a genus **(C)** and family level **(D)**. Communities of high and low FCR birds were significantly different based on Canberra distance at a genus (*p* = 7E^-5^) or family level (*p* = 3.9E^-4^).

**FIGURE 4:**
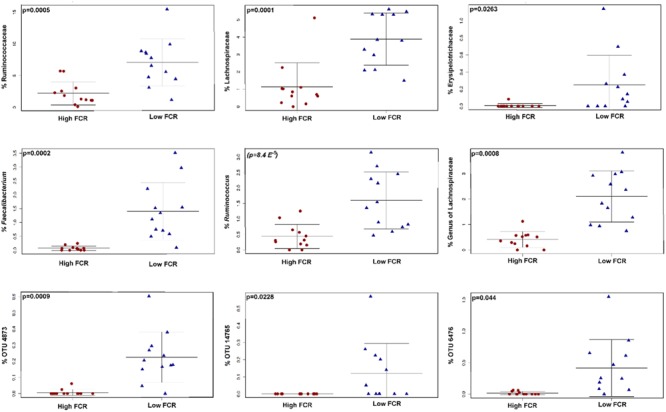
**The taxa responsible for differences in high and low FCR cecal communities in Trial 1.** The figure shows family **(top row)**, genus **(middle row)** and OTU level **(bottom row)**. A table with all differentially abundant phylotypes is given in Supplementary Table S1. The closest cultured strain (EzTaxon database) to OTU 4873 was *F. praustnizii* [ATCC 27768(T)] with 93.7% pairwise similarity, OTU 14765 was closest to *Clostridium spiroforme* [DSM 1552(T)] with 99.6% pairwise similarity to the type strain and out 6476 was closest to *C. lactatifermentans* (89.76%).

QIIME-based alpha diversity statistical analysis was performed using a non-parametric, two-sample *t*-test and 1000 Monte Carlo permutations to inspect statistical significance of Chao1, observed species, Shannon and Simpson alpha metrics. In Trial 1, the birds with good FCR showed higher diversity with significantly higher chao1 (*p* = 0.0021, **Figure 3A**) and observed species alpha metrics (*p* = 3E^-4^) than the birds with poor FCR. They also displayed significantly higher richness and evenness indices than poor FCR birds at a family and a genus level (Supplementary Figure S4). There was strong separation between poor (high) and good (low) FCR birds in the phylotypes present, measured as Unweighted UniFrac (Adonis *p* = 3E^-4^, **Figure 3B**) while there were no differences in phylotype abundance in Weighted Unifrac distance (*p* = 0.2526). The differences in community structure were evident at both genus (*p* = 7E^-5^) and family level (*p* = 3.9E^-4^) using Canberra distance (**Figures 3C,D**). Significance analysis was performed (*t*-test, QIIME), comparing the 12 best and poorest FCR birds. The differences were prominent at a family and genus level (**Figure 4**). Three families were responsible for high community differences at a family level: *Lachnospiraceae* (*p* = 1.1E^-4^), *Ruminococcaceae* (*p* = 5.09E^-4^), and *Erysipelotrichaceae* (*p* = 0.026) (**Figure 4**). At the genus level, genera significantly more abundant in good FCR included *Ruminococcus, Faecalibacterium, Clostridium* and two unknown genera from the *Lachnospiraceae* family, showing differential abundance with *p*-values ranging from 0.02 to 8E^-5^ and 2.5 to 20.1-fold higher in better performing birds (Supplementary Table S1). Genus *Faecalibacterium* was 20.1 times more abundant in good FCR birds, while OTU18285, 16.8 times more abundant in good FCR birds (*p* = 0.0016), aligned with *Faecalibacterium prausnitzii* strain ATCC 27768(T) with 94.74% identity (EzTaxon database). In Trial 1 21 differentially abundant OTUs were identified (Qiime *t*-test, *p* < 0.05) between high and low FCR birds. A table with the major significantly differential phylotypes at family, genus, and OTU levels (*p* < 0.01) is provided (Supplementary Table S1).

Correlations between phylotypes on each taxonomic level vs. FCR values were then inspected. This analysis investigated correlation in all birds between all of the phylotypes (at family, genus and species level) vs. FCR. At the family level, *Lachnospiraceae* showed the most significant (*p* = 3.6E^-4^) negative correlation with FCR values (Supplementary Figure S5) followed by two other significantly negatively correlated families *Erysipelotrichaceae* (*p* = 0.0053) and *Ruminococcaceae* (*p* = 0.0259). Negative correlation with FCR values indicates a positive effect on performance as lower FCR indicates better performance. Significant correlations between taxa, at a family, genus and OTU level, are shown in Supplementary Table S2.

**FIGURE 5:**
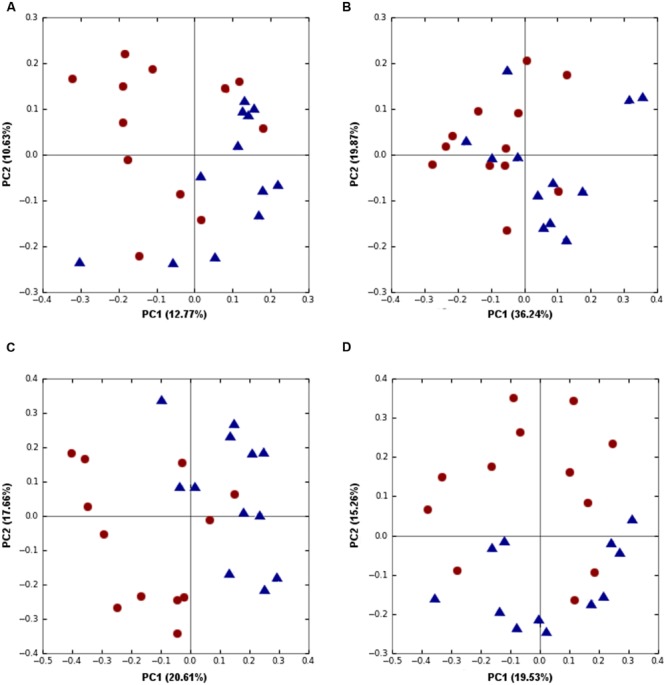
**Trial 2 beta diversity was significantly different between high (red) and low (blue) FCR birds microbial communities. (A)** Unweighted (*p* = 0.0019) and **(B)** Weighted Unifrac (*p* = 0.0072) at an OTU level as well as Canberra beta diversity at a **(C)** genus (*p* = 0.0055) and **(D)** family (*p* = 1.8E^-4^) levels were separating high and low FCR birds.

**FIGURE 6:**
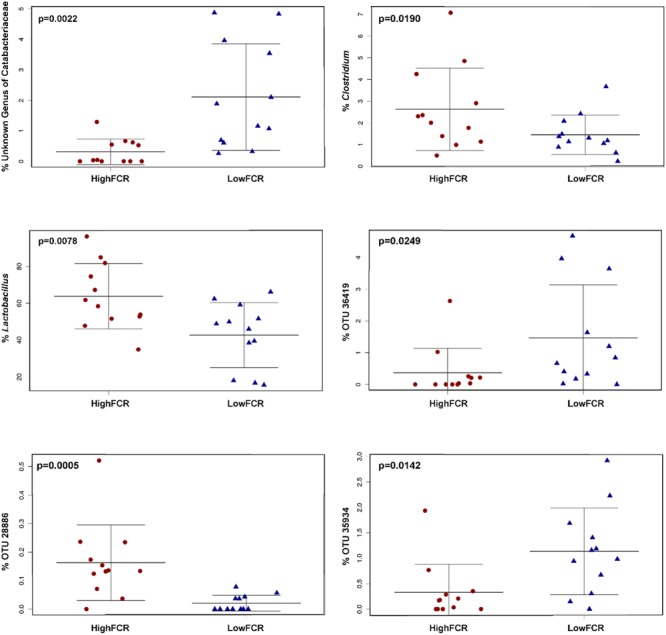
**Some of the significantly (FCR) differential genera and OTUs in Trial 2.** Genus *Clostridium* significantly correlated with poor performing (high FCR) birds is of the lineage *Clostridiaceae/Clostridium*. The closest cultured strain to OTU36419 was *Ruminococcus albus* (similarity 90.06%), to OTU28886 *Lactobacillus reuteri* (98.98%) and to OTU35934 *C. cellobioparum* (83.13%).

The Trial 2 microbiota community was very different to that observed in Trial 1 and very little overlap was found between phylotypes associated with FCR, even at a family level. There were no significant differences in alpha diversity measures between good and poor FCR bird microbial communities, unlike in Trial 1. However, strong differences were observed in Unweighted (*p* = 0.0019) and Weighted Unifrac (*p* = 0.0072) at an OTU as well as at higher taxonomic ranks, using Canberra distance, at genus (*p* = 0.0055) and family (*p* = 1.8E^-4^) levels (**Figures 5A–D**). The order responsible for most of the differences was the same as in Trial 1 – *Clostridiales*, however, instead of families *Lachnospiraceae* and *Ruminococcaceae*, in Trial 2 the significantly differential families were *Catabacteriaceae* and an unknown family of order *Clostridiales* (**Figure 6**, Supplementary Table S1). Surprisingly, the genus *Lactobacillus* was enriched (*p* = 0.0078) in poor FCR birds (**Figure 6**, Supplementary Table S1). Correlation analysis included all birds from the trials and matched the results from significance analysis (Supplementary Table S2).

Few differences were noted in the microbiota of good and poor FCR birds in Trial 3 compared to the other two trials. There was no significant difference in alpha or beta diversity measures at any of the taxonomic levels. There were no families or genera significantly different in abundance between good and poor birds nor were there any significant correlations at these levels. At an OTU level, four OTUs were significantly (*p* < 0.01) different in abundance between good and poor FCR birds. The OTUs enriched in poor FCR birds belonged to *Lactobacillus* and *Faecalibacterium* genera (Supplementary Table S1).

**FIGURE 7:**
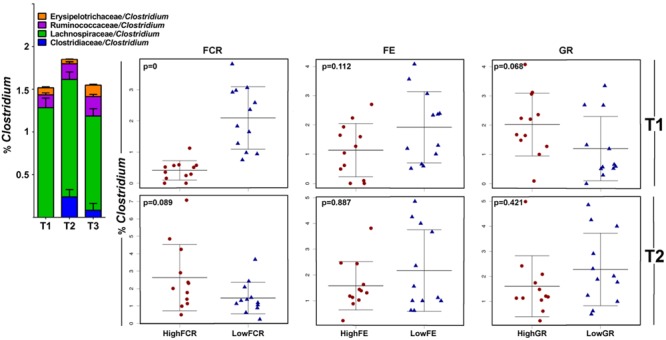
**Different members of the genus *Clostridium* correlate with growth performance in different trials.** In this analysis all genera listed as *Clostridium* were merged and the correlation of total *Clostridium* abundance is shown in stripcharts for Trial 1 **(top row)** and Trial 2 **(bottom row)**; there were no differences in Trial 3. *Clostridium* species had lineages split between families *Clostridiaceae, Ruminococcaceae, Lachnospiraceae*, and *Eryspelotrichaceae*. The trial 1 *Clostridium* community, with no members of the *Clostridiaceae*/*Clostridium* lineage (blue on the barchart above), correlated with improved FCR and slightly reduction in FE and increased GR. In Trial 2 (and more so in Trial 3), the *Clostridiaceae* lineage of *Clostridium* gave non-significant differences in the opposite FCR direction.

Families *Lachnospiraceae, Erysipelotrichaceae*, and *Ruminococcaceae* were found to be significantly negatively correlated with FCR values. Since reduction of FCR (feed needed per kg of weight) is the aim of the animal production industries, these families are identified as a source of candidate probiotic isolates with potential to be used for performance enhancement. All three families owed their significance to the ambiguously classified genus *Clostridium*, which appeared in 4 different lineages (**Figure 7**). Significant *Lachnospiraceae*/*Clostridium* OTUs were comprised of sequences most similar to *Clostridium lactatifermentans* (95.35%), *Erysipelotrichaceae*/*Clostridium* to *C. spiroforme* (93%) and *Ruminococcaceae/Clostridium* to *C. leptum* (91.6%).

Trial 3 lacked significance at higher levels and showed equivocal results at an OTU level with a number of OTUs with similar phylogenetic classification differentially abundant in both good and bad FCR birds. There were 17 *Lactobacillus* OTUs among the differentially abundant (*p* < 0.05), however, the genus *Lactobacillus*, as a whole, was unchanged suggesting community shifts within the *Lactobacillus* genus (Supplementary Figure S6).

### Phylotypes Associated with Efficiency of Energy Extraction from Feed

The differences between high and low AME birds were significant (*p* < 0.0001) in each of the three trials based on a non-parametric Mann–Whitney *U* test (Supplementary Figure S1). The data analysis and comparisons of the three trials pointed to Trial 2 as the trial displaying the highest, however, still moderate, microbiota differences between high and low AME birds, followed by Trial 3, and with little response recorded in Trial 1, compared at all phylogenetic levels.

Differences in alpha diversity were only observed in Trial 2 where the better energy assimilating, high AME, birds had microbiota with lower dominance (*p* = 0.007) and higher equitability, Shannon and Simpson indices (*p* = 0.0236, *p* = 0.0448, and *p* = 0.0064, respectively). However, there were no differences observed in alpha diversity between high and low AME birds in Trials 1 and 3 caecal microbiotas.

No differences in overall community structure were detected between high and low AME birds in any of the three trials using Weighted and Unweighted UniFrac and Anosim statistics indicating that differences in AME are not driven by overall total community shifts in either alpha or beta diversity. A few OTUs were found to be differentially abundant between high and low AME birds (Supplementary Table S3) with a Rumininocaccaceae related OTU 16-fold more abundant in the high AME birds in Trial 2. There were no family or genus level phylotypes significantly (*p* < 0.05, *r* > 0.3) correlated with AME but at the OTU level *Lactobacillus. reuteri* OTU28886, (*p* = 8.28E^-6^, *r* = -0.50, Supplementary Table S4) and six other OTUs identified as *L. reuteri* were all significantly negatively correlated (*p* < 0.05, *r* = -0.24 to -0.34) with energy extraction from feed. *L. crispatus* OTU 15229 abundance was positively correlated (*p* = 0.0076, *r* = 0.33) with AME values.

### Correlations Between Bacterial Abundance and Gain Rate and Feed Eaten

**FIGURE 8:**
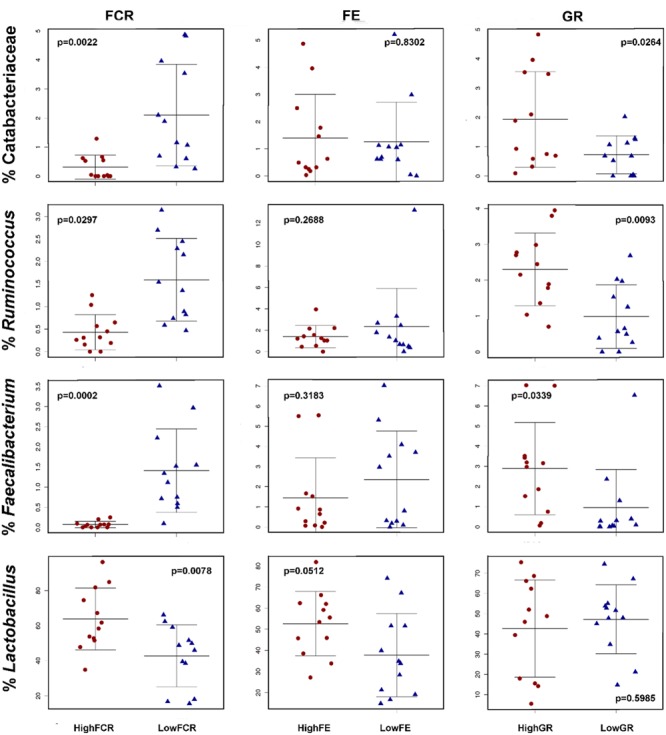
**Feed conversion ratio associated OTUs have variable correlations with FE and GR performance.** Bacteria correlated in abundance with good (low) FCR levels were generally also correlated with good (high) GR levels but showed no significant correlation with either high or low FE. **(Top row)** the family *Catabacteriaceae* from Trial 2. **(Second row)** *Ruminococcus* from Trial 1. **(Third row)** *Faecalibacterium* from Trial 1. **(Fourth row)** in Trial 2 the *Lactobacillus* genera was associated with poor FCR performance but showed no significant correlation with either FE or GR levels.

There were no significant differences in overall community structure, shown as either alpha or beta diversity differences between extremes of GR or FE in any of the three trials. Both GR and FE extreme birds showed few individual phylotypes that were significantly different in each trial (Supplementary Tables S5–S8). We noted that in trials 1 and 2 the same families and genera enhanced in good FCR birds were also significantly associated with good GR (**Figure 8**, Supplementary Tables S1 and S5). This was not reproduced with FE; phylotypes involved did not overlap with phylotypes associated with FCR extremes. This observation indicates that different phylotypes were correlated with GR and performance compared to those that correlated with FE (**Figure 8**).

### Random Forest Data Modeling

Phylotypes associated with high performance over a combination of performance measures were investigated by constructing a ranked list of all birds, in each trial separately, ranking low to high FCR and high to low AME birds. The combined AME and FCR ranks with low values were used to identify the birds with a combination of desirable high AME and low FCR values. These birds are the most efficient in extracting energy from food (high AME) and converting feed to body weight (low FCR). They were identified as AME_FCR_Good. The birds with high rank values showed the opposite trend of low AME and high FCR; they were classified as AME_FCR_Bad. Upon inspecting 50 Random Forest prediction trees, no OTUs were identified as clearly implicated in differentiating the AME_FCR_Good birds from the other birds that were also identified in the correction analysis carried out independently for the AME or FCR single performance variable analysis above. However, the Random Forest data analysis approach did identify *F. prausnitzii, Bacteroides fragilis*, and members of *Ruminococcus* genus repeatedly associated with good performance in a range of prediction trees, while members of *Gammaproteobacteria* and members of the genus *Clostridium* were associated with the undesirable AME_FCR_Bad birds (Supplementary Figure S7). *F. prausnitzii* was identified in the significance analysis of Trial 1 as being more highly abundant in the good FCR birds so the Random Forest analysis has extended this to a more general finding over all the birds and in combined AME_FCR good birds.

## Discussion

In all three trials the average growth rate and feed conversion of the chickens met or exceeded the breeder’s expectations (Cobb-Vantress, 2013), however, within each trial there were differences in the performance parameters of birds and so relatively high and low performance birds could be identified. We have previously reported large variations between the gut microbiota in these three separate flocks, as well as considerable between-bird variation within each flock (Stanley et al., 2013b). The current analysis aimed to determine whether, in the face of this sort of microbiota variation, there were common changes in the phylotype profiles that characterized the enhanced performance birds in the different trials. In each of the three trials there where bacterial phylotypes that were differentially abundant between the high and low performance birds. However, it was found that some phylotypes associated with high performance in one trial were completely absent in another trial. With the variation in background microbiota between trials there were no OTUs that were consistently associated with good or bad performance across all three trials.

In the present study several probiotic candidates and taxonomic groups with potential benefits to performance were identified. In the first trial, there was a strong dominance of *Clostridia* influence. The *Clostridia* are a highly polyphyletic class of *Firmicutes* converged from multiple ancestors thus believed to be in need of reclassification. There are over 140 annotated and described *Clostridium* species, however, 16S rRNA gene sequencing resulted in a major revision of the genus (Stackebrandt et al., 1999), describing many *Clostridium* species as new genera and associating many others to different known genera. Despite major revisions the *Clostridium* species occupy different positions within the 16S rDNA phylogenetic tree (Stackebrandt et al., 1999; Biddle et al., 2013). Many of the *Clostridium* species moved to different families kept the genus name *Clostridium* adding to the taxonomic confusion. Moreover, different databases take different views on the issue and display different lineages for the same species. *Clostridium* species are now referred to as a number of gram positive and negative bacteria, with some not even being anaerobes (Fak and Backhed, 2012). In 2013 renaming of ambiguously named *Clostridium* species was suggested (Yutin and Galperin, 2013). In our Trial 1, *Clostridium* species were classified using GreenGenes database, to 4 different families (*Clostridiaceae, Ruminococcaceae, Lachnospiraceae*, and *Erysipelotrichaceae*). Species of *Clostridium*, from family *Clostridiaceae*, were implicated in poor performance, however, the species of *Clostridium* classified as *Lachnospiraceae, Ruminococcaceae*, and *Erysipelotrichaceae*, based on 16S rRNA gene sequence, showed significant positive correlation between relative abundance and good FCR performance at a family, genus and species level. *Lachnospiraceae* and *Ruminococcaceae* are associated with gut health through short chain fatty acid (SCFA) production and degradation of plant materials (Biddle et al., 2013) while *Erysipelotrichaceae* family members were implicated in weight gain in a human study where they were present only in obese individuals (Zhang et al., 2009).

The relative abundance of the *Lachnospiraceae* family as a whole was positively correlated with good FCR performance. A *Lachnospiraceae* family classified genus *Clostridium*, with members closest (95.35%) to *C. lactatifermentans*, was significantly increased in low FCR birds (4.7-fold). It was recently suggested that the genus should be renamed to *Lachnoclostridium* (Yutin and Galperin, 2013). *C. lactatifermentans* was isolated from the caeca of chicken and described by van der Wielen et al. (2002b). The same group has investigated the use this lactate-fermenting strain as a chicken probiotic. They demonstrated that *C. lactatifermentans* inhibited the growth of *Salmonella enterica* in batch culture simulating caecal conditions (van der Wielen et al., 2002a). The authors argued that the ability of *C. lactatifermentans* to ferment lactate to SCFAs, namely acetate, propionate and traces of butyrate and isovalerate, ought to be beneficial to the host (van der Wielen et al., 2002a,b,c). SCFAs are known to be important in colonic health and reduce the risk of inflammatory bowel disease, irritable bowel syndrome, cancer and cardiovascular disease in humans (Hijova and Chmelarova, 2007). SCFAs can increase the growth of beneficial bacteria, *Lactobacillus* and *Bifidobacteria* (Roy et al., 2006), and are a major energy source in the intestine. Lactic acid produced by *Lactobacillus* strains can be converted to the most beneficial SCFAs such as acetate, propionate and butyrate by *C. lactatifermentans*. The vast majority of produced acetate is readily transported to the liver and metabolised. The liver also uses residual butyrate and propionate for gluconeogenesis. Butyrate is the main energy source for caecal and colonic epithelial cells. Acetate is an energy source for muscle (reviewed by Hijova and Chmelarova, 2007) and propionate and butyrate regulate the expression of the *FFAR3* gene, which leads to fat deposition in adipocytes (Vangaveti et al., 2010). Thus, there are good theoretical reasons why the increased abundance of *C. lactatifermentans* could potentially improve colonic and liver heath of the chicken, stimulate muscle and control fat deposits via the production of SCFAs. It seems likely that *C. lactatifermentans* within the microbial community, could increase performance via conversion of *Lactobacillus* metabolites to beneficial SCFAs. Similarly, the whole butyrate-producing *Lachnospiraceae* family potentially have beneficial effects.

The *C. spiroforme* related OTU with the strongest correlation with FCR performance was 99.6% identical to the type strain, while the most abundant *C. spiroforme* OTU, comprising most of the microbiota contribution from the *Erysipelotrichaceae* family, was only 93% similar to the type strain. *C. spiroforme* was recently transferred (reviewed in Yutin and Galperin, 2013) to the family *Erysipelotrichaceae* in the class *Erysipelotrichi*, within the new genus, *Erysipelatoclostridium* (Yutin and Galperin, 2013). *C. spiroforme* is often cited in the literature for toxin production and is implicated in gastrointestinal issues (Perelle et al., 1997). However, the most abundant strain driving this genus significance in FCR, in our data is only 93% similar to the type strain thus allowing for the possibility of a non-toxic relative being involved in broiler performance especially with the knowledge of the role of *Erysipelotrichaceae* in weight gain (Zhang et al., 2009).

Among other candidates as performance enhancing probiotics, one of the most exciting is *F. prausnitzii*. Newly emerging evidence indicates that *F. prausnitzii* has an important role in establishing and maintaining healthy metabolism, SCFA production and appropriate development of the immune system in humans. Reduction in the abundance of *F. prausnitzii* is linked to numerous diseases including diabetes, colitis, IBD, dysbiosis and immunocompromised states (reviewed in Miquel et al., 2013). This bacterium was identified as correlated with good performance in Trial 1 and the extended analysis with the Random Forest approach indicated that it was more broadly indicative of high performance birds rated on joint FCR and AME rankings across the three trials. Although clearly of interest there are difficulties in developing this bacterium as a probiotic as it is a strict anaerobe and it is not clear whether it can be economically grown and delivered on a large scale.

We identified some members of the *Lactobacillus* genus as undesirable for overall performance, mostly due to an increase in FE. The link between *Lactobacillus* and increased appetite and feed consumption has been shown in humans. Although there are strains of *Lactobacillus* known to improve performance, there are numerous strains retailed as weight loss probiotics and others with reported ability to reduce obesity (Fak and Backhed, 2012). The same finding of a negative influence of some *Lactobacillus* strains on performance has been previously reported (Torok et al., 2011). Moreover, different strains of the same species may act in the opposite manner: *L. reuteri* L6798 was associated with weight gain, whereas *L. reuteri* ATCC PTA 4659 was associated with weight loss in mice (Fak and Backhed, 2012). In Trial 3, most of the differential OTUs were *Lactobacillus* species correlated with both good and poor performance, while the genus *Lactobacillus*, as a whole, remained unchanged in relative abundance, suggesting a number of strains have conflicting effects on performance.

Our data suggest that the use of *Lactobacillus* isolates as probiotics must be approached with caution. Although some *Lactobacillus* OTUs are correlated with superior performance other OTUs, even of the same predicted species, are correlated with poor performance. There are other valid probiotic candidates emerging from this microbiota analysis. From the present dataset, beneficial Clostridia members, involved in plant material degradation, cellulose utilization and SCFA production are associated with growth performance. This is in agreement with previously published 16S based studies in broiler performance (Torok et al., 2008, 2011; Stanley et al., 2012, 2013a).

Significant variability in microbiota requires development of new strategies in culturing, isolate selection and *in vivo* testing in order to identify isolates that could have utility as probiotics. Strains will need to be chosen on the basis that when introduced to newly hatched chickens, they colonize in high abundance in the face of different background microbiotas and thus improve performance regardless of other organisms in the gut population. The ability to colonize in diverse microbiota backgrounds is likely to be a key attribute of probiotics that are able to reliably deliver desired benefits to the host.

## Author Contributions

RM, RH, and MG designed the study, RH and MG conducted the animal trial work. RM and DS prepared microbiome DNA and generated 16S amplicons for sequencing. DS carried out the bioinformatics analysis of the data and drafted the manuscript. RM and RH edited the manuscript. All authors approved the final version of the manuscript.

## Conflict of Interest Statement

The authors declare that the research was conducted in the absence of any commercial or financial relationships that could be construed as a potential conflict of interest.
