# Supplementary material for: Bacteria within the Gastrointestinal Tract Microbiota Correlated with Improved Growth and Feed Conversion: Challenges Presented for the Identification of Performance Enhancing Probiotic Bacteria
Source: Front Microbiol. 2016 Feb 19;7:187. doi: 10.3389/fmicb.2016.00187 (PMC4760072; doi:10.3389/fmicb.2016.00187)
Supplement: Supplementary file 1 [file Data_Sheet_1.PDF]

Bacteria within the gastrointestinal tract microbiota correlated with improved growth and feed conversion: Challenges presented for the identification of performance enhancing probiotic bacteria

Running Title: Microbiota and broiler performance

Dragana Stanley[1,2,], Robert J. Hughes[2,3,4], Mark S. Geier[5], and Robert J. Moore[2,6,7,8,*]

[1]Institute for Future Farming Systems, Central Queensland University, Rockhampton, Queensland, Australia.
[2]Poultry Cooperative Research Centre, University of New England Armidale, New South Wales, Australia.
[3]Pig and Poultry Production Institute, South Australian Research and Development Institute, Roseworthy, South Australia, Australia.
[4]School of Animal and Veterinary Sciences, The University of Adelaide, Roseworthy, South Australia, Australia.
[5]Research and Innovation Services, The University of South Australia, Mawson Lakes, South Australia, Australia.
[6]Australian Animal Health Laboratory, CSIRO, Geelong, Victoria, Australia.
[7]School of Science, RMIT University, Bundoora , Victoria, Australia.
[8]Department of Microbiology, Monash University, Clayton, Victoria, Australia.

[*] **Correspondence:**
Professor Robert Moore
Host-Microbe Interactions Laboratory
School of Science
RMIT University, Bundoora West Campus
Building 223, Level 1, Rm28D
Bundoora, Victoria 3083, Australia
Email: rob.moore@rmit.edu.au
Phone: +61 3 9925 7580

Supplementary Figures

Figure S1

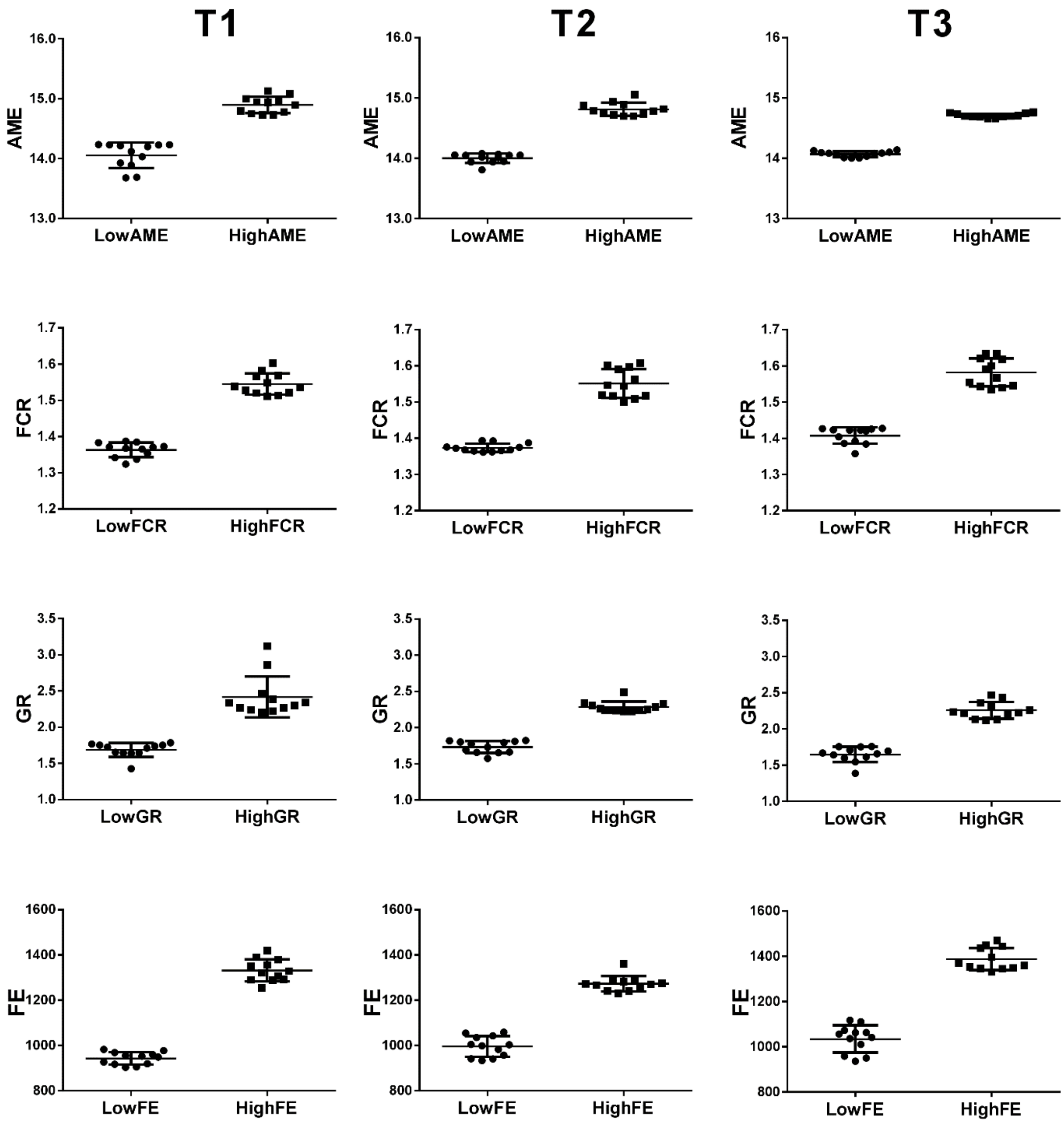

Figure S1| Best and worst performing 12 birds of the flock. The birds were differential in AME, FCR, GR and FE based on nonparametric Mann-Whitney U test, in all 3 trials (T1-T3). All p-values were <0.0001.

Figure S2

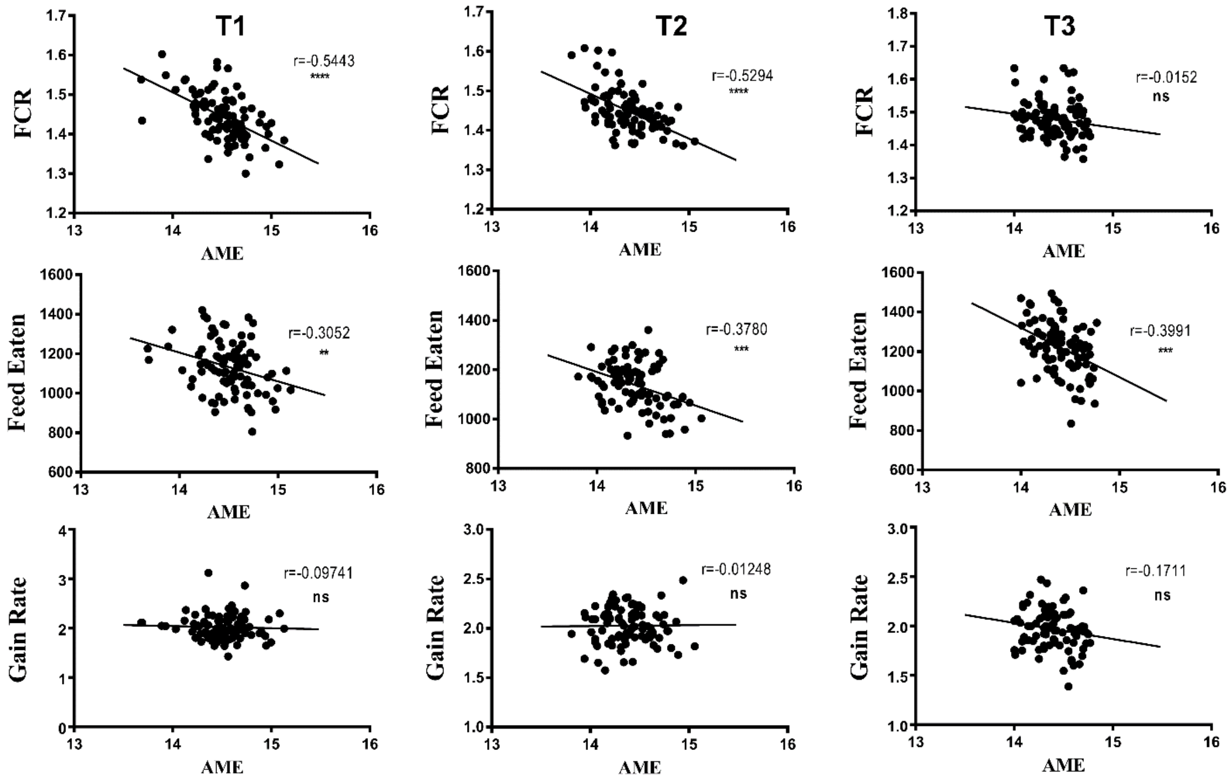

Figure S2 | Correlations of AME against FCR, FE and GR. The three trials are given in separate columns, Trial 1 in the left column, Trial 2 in the middle and T3 in the right column. The birds with higher AME values corresponding to better efficiency in energy extraction are likely to have lower (better) FCR, mostly due to eating less feed while having no change in gain rate. Figure was generated using animal trial data and GraphPad Prizm software. P-value significance level and correlation coefficient (r) are based on Pearson correlation analysis between the two variables compared.

Figure S3

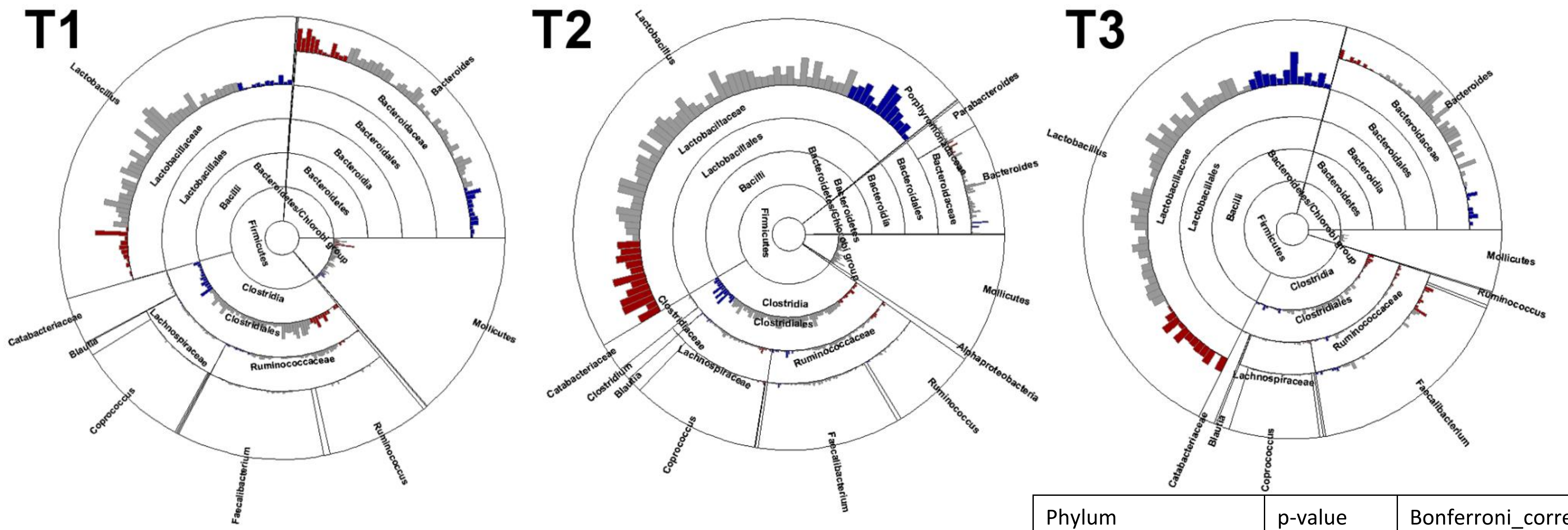

Phylum	p-value	Bonferroni_corrected
Firmicutes	$5.40E^{-10}$	$2.70E^{-09}$
Tenericutes	$4.52E^{-09}$	$2.26E^{-08}$
Bacteroidetes	$5.12E^{-08}$	$2.56E^{-07}$
Proteobacteria	$2.05E^{-06}$	$1.03E^{-05}$

Figure S3 | Taxonomy profile of the 3 trials shows significant differences at phylum level. The graph was created by importing a QIIME biom table for each trial into MEGAN software (Huson et al., 2007). The size of the bars is proportional to the taxa abundance in each sample. Samples with

highest FCR are coloured red, lowest FCR blue and the remaining samples are grey. The three flocks were dominated by different phyla (Stanley et al., 2013).

FigureS4

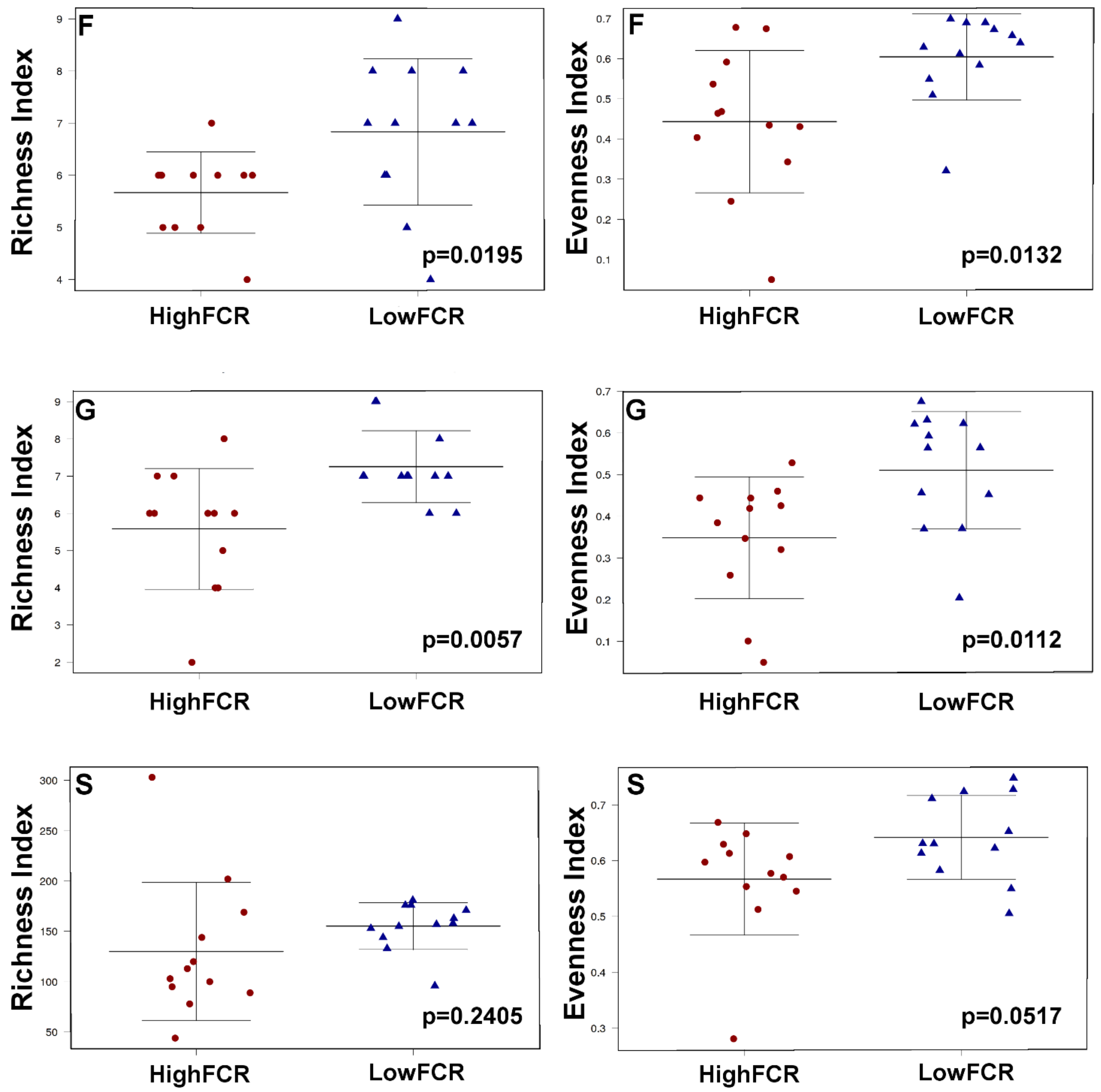

Figure S4 | Alpha diversity measures, **Trial 1:** Alpha diversity, expressed as Richness and Evenness index, was differential between high and low FCR birds microbial communities at a family (row 1-labeled F) and genus (row 2 labelled G), but not at a species (OTU) level (row 3 labelled S).

Figure S5

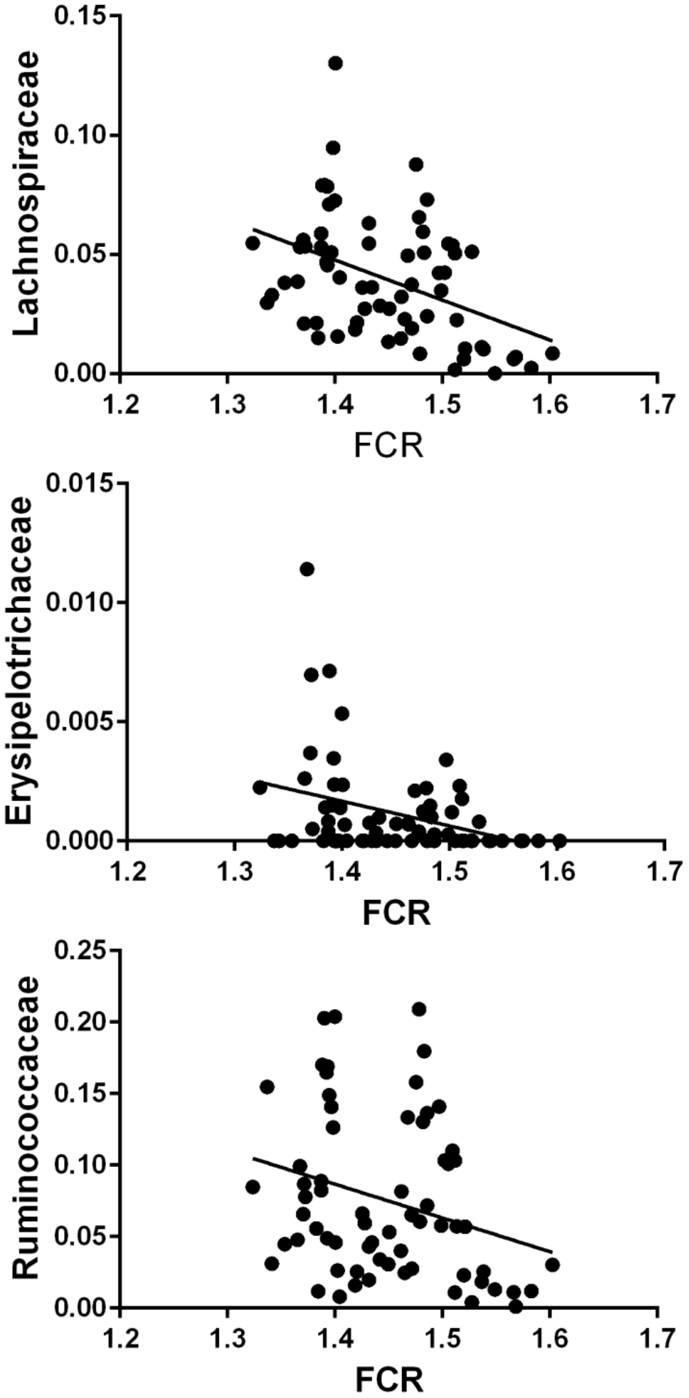

Figure S5 | Trial 1; three families showed significant negative Pearson correlation with FCR. *Lachnospiraceae* ($p=3.6E^{-4}$), *Erysipelotrichaceae* ($p=5.0E^{-4}$) and *Ruminococcaceae* (p=0.0259).

Figure S6

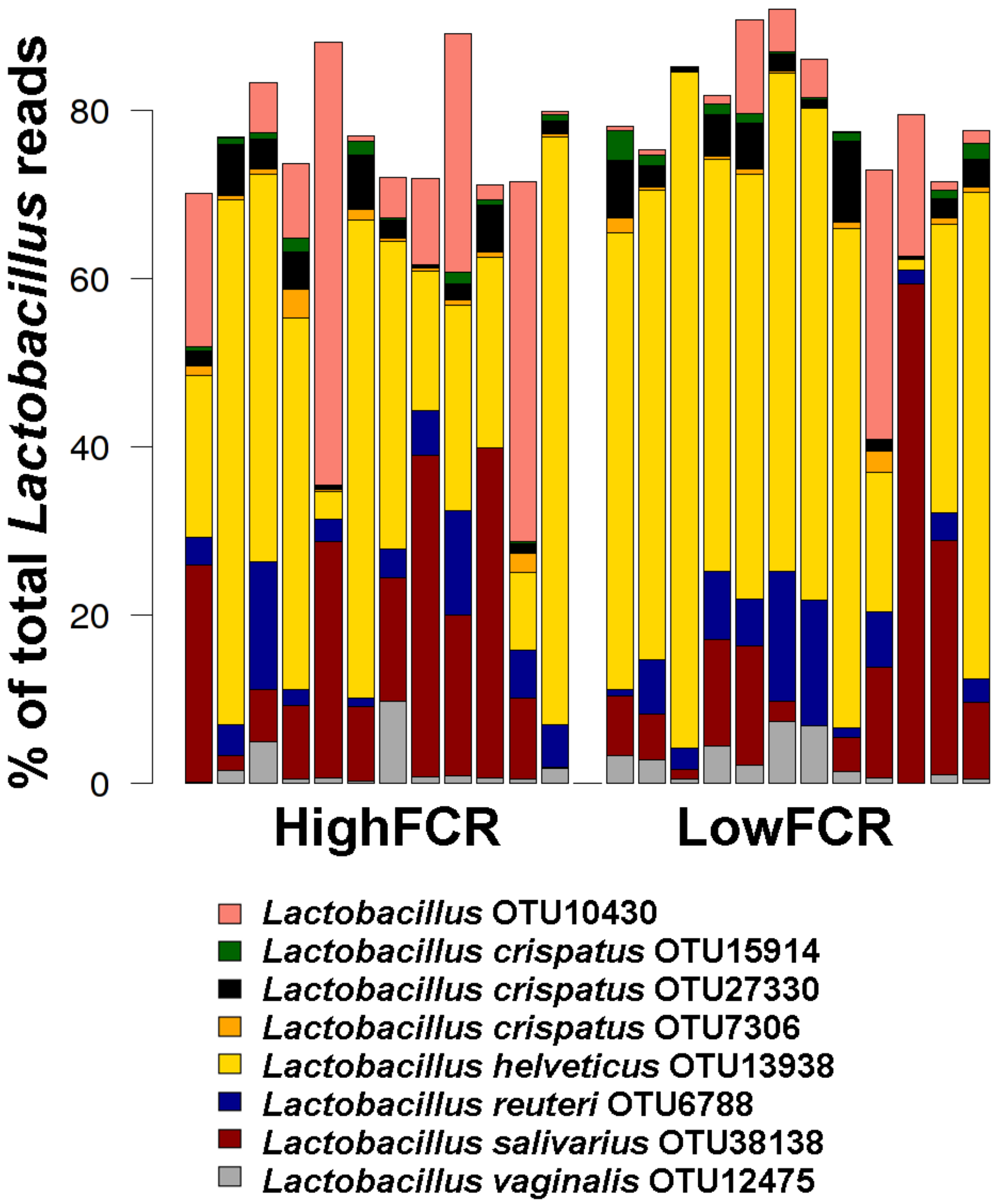

Figure S6 | *Lactobacillus* profile shifts in high and low FCR birds in Trial 3. There were 13 *Lactobacillus* OTUs more abundant ($p<0.05$) in good FCR birds and 5 others more abundant in poor FCR birds. Regardless of *Lactobacillus* OTUs dominating list of differential OTUs in trial 3, genus *Lactobacillus* was not significantly differentially abundant between high and low FCR birds indicating that shifts were occurring at the species level in both directions. In trial 2 *Lactobacillus* increased FCR by increasing feed eaten (Figure 8).

Figure S7

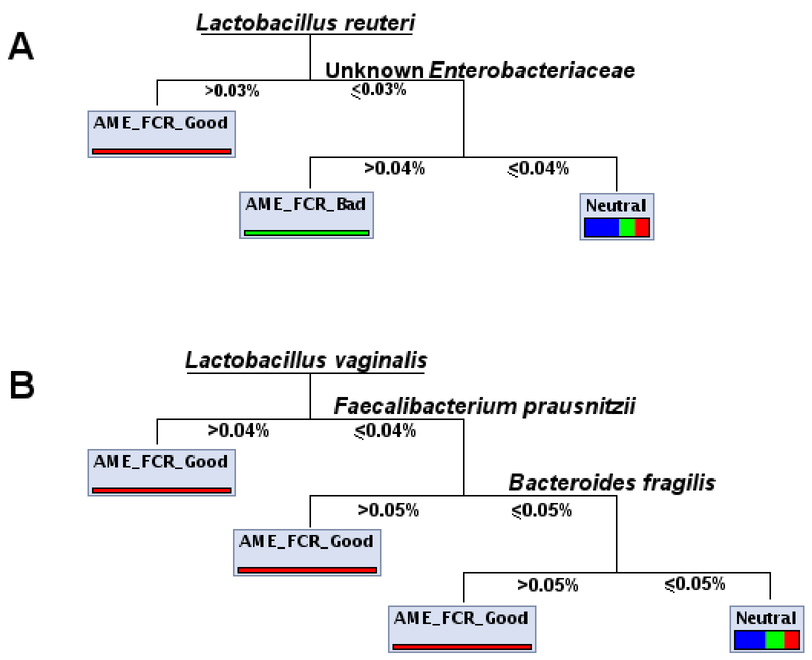

Figure S7 | Random Forest data modelling. A Random Forest algorithm was used to predict association of OTUs with birds that had both good AME and good FCR performance and also with undesirable bird trait - bad AME and bad FCR values. RapidMiner software was used to generate 50 predication trees, two of which are presented as A and B above. Each prediction was inspected and OTUs identified as associated with good performance included a number of OTUs identified as *Faecalibacterium prausnitzii*, *Bacteroides fragilis* and *Ruminococcus* species. Members of *Gammaproteobacteria* and genus *Clostridium* were repetitively associated with bad AME and bad FCR. Both these trees show *Lactobacillus* OTUs associated with good performance but other trees had members of the genus *Lactobacillus* whose abundance was positively predictive of bad performance.

Supplementary Tables

Please note that supplementary tables present only data with p<0.01 due to the size of p<0.05 data for all 8 tables presented.

Table S1 | Different levels of taxa significantly (QIIME t-test, p<0.01) differentially abundant between microbial communities sampled from the 12vs. 12 extreme birds based on FCR. High indicates that taxa is present only in high **FCR** birds.

**TRIAL 1**	GreenGenes taxonomy	p-value	FoldGood
FAMILY	Firmicutes/Clostridia/Clostridiales/Lachnospiraceae	0.0001	3.41
FAMILY	Firmicutes/Clostridia/Clostridiales/Ruminococcaceae	0.0005	3.22
FAMILY	Tenericutes/Erysipelotrichi/Erysipelotrichales/Erysipelotrichaceae	0.0263	36.57
GENUS	Firmicutes/Clostridia/Clostridiales/Lachnospiraceae/*Clostridium*	8.04E-05	4.76
GENUS	Firmicutes/Clostridia/Clostridiales/Ruminococcaceae/*Faecalibacterium*	0.0002	20.14
GENUS	Firmicutes/Clostridia/Clostridiales/Lachnospiraceae/unknown	0.0008	3.80
GENUS	Firmicutes/Clostridia/Clostridiales/Ruminococcaceae/unknown	0.0092	2.51
GENUS	Firmicutes/Clostridia/Clostridiales/Lachnospiraceae/*Ruminococcus*	0.0132	3.83
GENUS	Firmicutes/Clostridia/Clostridiales/Ruminococcaceae/*Ruminococcus*	0.0297	3.60
denovo4873	Firmicutes/Clostridia/Clostridiales/Ruminococcaceae/*Faecalibacterium*	0.0009	22.00
denovo13839	Tenericutes/Mollicutes/RF39	0.0011	-3.89
denovo18285	Firmicutes/Clostridia/Clostridiales/Ruminococcaceae/*Faecalibacterium*	0.0016	16.86
denovo6476	Firmicutes/Clostridia/Clostridiales/Lachnospiraceae/*Clostridium*	0.0044	15.80
**TRIAL 2**	GreenGenes taxonomy	p-value	FoldGood

FAMILY	Firmicutes/Clostridia/Clostridiales/unknown	6.11E-05	2.83
FAMILY	Firmicutes/Clostridia/Clostridiales/Catabacteriaceae	0.0022	6.79
FAMILY	Firmicutes/Bacilli/Lactobacillales/Lactobacillaceae	0.0079	-1.49
GENUS	Firmicutes/Clostridia/Clostridiales/unknown	6.11E-05	2.83
GENUS	Firmicutes/Clostridia/Clostridiales/Catabacteriaceae/unknown	0.0022	6.79
GENUS	Firmicutes/Bacilli/Lactobacillales/Lactobacillaceae/*Lactobacillus*	0.0079	-1.49
denovo28886	Firmicutes/Bacilli/Lactobacillales/Lactobacillaceae/*Lactobacillus/Lactobacillus reuteri*	0.0005	-8.33
denovo30978	Firmicutes/Bacilli/Lactobacillales/Lactobacillaceae/*Lactobacillus*	0.0017	-4.75
denovo14829	Firmicutes/Bacilli/Lactobacillales/Lactobacillaceae/*Lactobacillus/Lactobacillus reuteri*	0.0020	-14.00
denovo23619	Firmicutes/Clostridia/Clostridiales	0.0032	Low
denovo721	Firmicutes/Bacilli/Lactobacillales/Lactobacillaceae/*Lactobacillus/Lactobacillus vaginalis*	0.0063	High
denovo39924	Firmicutes/Bacilli/Lactobacillales/Lactobacillaceae/*Lactobacillus*	0.0064	-7.75
denovo14082	Firmicutes/Bacilli/Lactobacillales/Lactobacillaceae/*Lactobacillus/Lactobacillus reuteri*	0.0070	-3.83
denovo27448	Firmicutes/Bacilli/Lactobacillales/Lactobacillaceae/*Lactobacillus*	0.0086	-14.00
**TRIAL 3**	GreenGenes taxonomy	p-value	FoldGood
denovo4280	Firmicutes/Bacilli/Lactobacillales/Lactobacillaceae/*Lactobacillus*	0.0031	High
denovo31256	Firmicutes/Clostridia/Clostridiales/Ruminococcaceae/*Faecalibacterium*	0.0046	-7.00
denovo24938	Firmicutes/Clostridia/Clostridiales/Ruminococcaceae/*Faecalibacterium*	0.0071	High
denovo22297	Firmicutes/Clostridia/Clostridiales/Ruminococcaceae	0.0088	2.52

Table S2 | Pearson correlations between different levels of taxa and **FCR** values in all birds from each trail.

**TRIAL 1**	GreenGenes taxonomy	p-value	r
FAMILY	Firmicutes/Clostridia/Clostridiales/Lachnospiraceae	0.0004	-0.43
FAMILY	Tenericutes/Erysipelotrichi/Erysipelotrichales/Erysipelotrichaceae	0.0053	-0.34

FAMILY	Bacteroidetes/Bacteroidia/Bacteroidales/Bacteroidaceae	0.0181	0.29
FAMILY	Firmicutes/Clostridia/Clostridiales/Ruminococcaceae	0.0260	-0.27
GENUS	Firmicutes/Clostridia/Clostridiales/Lachnospiraceae/*Clostridium*	0.0004	-0.42
GENUS	Firmicutes/Clostridia/Clostridiales/Lachnospiraceae/*Ruminococcus*	0.0094	-0.32
GENUS	Tenericutes/Erysipelotrichi/Erysipelotrichales/Erysipelotrichaceae/*Clostridium*	0.0118	-0.31
GENUS	Bacteroidetes/Bacteroidia/Bacteroidales/Bacteroidaceae/*Bacteroides*	0.0181	0.29
GENUS	Firmicutes/Clostridia/Clostridiales/Ruminococcaceae/*Faecalibacterium*	0.0217	-0.28
GENUS	Firmicutes/Clostridia/Clostridiales/Lachnospiraceae/unknown	0.0364	-0.26
denovo10379	Firmicutes/Clostridia/Clostridiales/Ruminococcaceae	0.0005	-0.42
denovo33809	Bacteroidetes/Bacteroidia/Bacteroidales/Bacteroidaceae/*Bacteroides/Bacteroides fragilis*	0.0008	0.40
denovo22831	Bacteroidetes/Bacteroidia/Bacteroidales/Bacteroidaceae/*Bacteroides/Bacteroides fragilis*	0.0020	0.37
denovo29625	Bacteroidetes/Bacteroidia/Bacteroidales/Bacteroidaceae/*Bacteroides/Bacteroides fragilis*	0.0030	0.36
denovo37095	Bacteroidetes/Bacteroidia/Bacteroidales/Bacteroidaceae/*Bacteroides/Bacteroides fragilis*	0.0037	0.35
denovo14765	Tenericutes/Erysipelotrichi/Erysipelotrichales/Erysipelotrichaceae/*Clostridium/Clostridium spiroforme*	0.0057	-0.34
denovo37572	Firmicutes/Clostridia/Clostridiales/Ruminococcaceae/*Faecalibacterium*	0.0092	-0.32
**TRIAL 2**	GreenGenes taxonomy	p-value	r
FAMILY	Firmicutes/Clostridia/Clostridiales/unknown	2.54E-05	-0.47
FAMILY	Firmicutes/Bacilli/Lactobacillales/Lactobacillaceae	0.0014	0.37
FAMILY	Firmicutes/Clostridia/Clostridiales/Catabacteriaceae	0.0016	-0.36
FAMILY	Tenericutes/Mollicutes/RF39/unknown	0.0212	-0.27
FAMILY	Proteobacteria/Alphaproteobacteria/unknown	0.0636	-0.22
FAMILY	Firmicutes/Clostridia/Clostridiales/Lachnospiraceae	0.0950	0.20
FAMILY	Firmicutes/Clostridia/Clostridiales/ClostridialesFamilyXIII.IncertaeSedis	0.1022	-0.19
GENUS	Firmicutes/Clostridia/Clostridiales/unknown	2.54E-05	-0.47
GENUS	Firmicutes/Bacilli/Lactobacillales/Lactobacillaceae/*Lactobacillus*	0.0014	0.37
GENUS	Firmicutes/Clostridia/Clostridiales/Catabacteriaceae/unknown	0.0016	-0.36
GENUS	Firmicutes/Clostridia/Clostridiales/Lachnospiraceae/*Coprococcus*	0.0019	0.36
GENUS	Firmicutes/Clostridia/Clostridiales/Lachnospiraceae/*Ruminococcus*	0.0031	0.34

GENUS	Tenericutes/Mollicutes/RF39/unknown	0.0212	-0.27
GENUS	Firmicutes/Clostridia/Clostridiales/Ruminococcaceae/*Ruminococcus*	0.0411	-0.24
denovo28886	Firmicutes/Bacilli/Lactobacillales/Lactobacillaceae/*Lactobacillus/Lactobacillus reuteri*	2.55E-07	0.56
denovo30978	Firmicutes/Bacilli/Lactobacillales/Lactobacillaceae/*Lactobacillus*	1.34E-06	0.53
denovo38034	Firmicutes/Bacilli/Lactobacillales/Lactobacillaceae/*Lactobacillus*	1.83E-06	0.53
denovo14829	Firmicutes/Bacilli/Lactobacillales/Lactobacillaceae/*Lactobacillus/Lactobacillus reuteri*	0.0002	0.43
denovo18242	Firmicutes/Clostridia/Clostridiales/Lachnospiraceae/*Coprococcus*	0.0009	0.38
denovo21177	Firmicutes/Bacilli/Lactobacillales/Lactobacillaceae/*Lactobacillus*	0.0010	0.38
denovo12890	Firmicutes/Clostridia/Clostridiales/Lachnospiraceae/*Ruminococcus/Ruminococcus torques*	0.0014	0.37
denovo26936	Firmicutes/Clostridia/Clostridiales	0.0039	-0.33
denovo21268	Bacteroidetes/Bacteroidia/Bacteroidales/Bacteroidaceae/*Bacteroides/Bacteroides fragilis*	0.0041	0.33
denovo13055	Firmicutes/Clostridia/Clostridiales/Ruminococcaceae	0.0062	0.32
denovo2375	Firmicutes/Clostridia/Clostridiales/Catabacteriaceae_	0.0072	-0.31
**TRIAL 3**	GreenGenes taxonomy	p-value	r
denovo22917	Firmicutes/Bacilli/Lactobacillales/Lactobacillaceae/*Lactobacillus/Lactobacillus reuteri*	0.0035	0.36
denovo12721	Firmicutes/Bacilli/Lactobacillales/Lactobacillaceae/*Lactobacillus*	0.0045	0.35
denovo9041	Firmicutes/Bacilli/Lactobacillales/Lactobacillaceae/*Lactobacillus/Lactobacillus crispatus*	0.0058	-0.34
denovo17075	Firmicutes/Clostridia/Clostridiales/Ruminococcaceae/*Ruminococcus*	0.0075	0.33
denovo6563	Firmicutes/Clostridia/Clostridiales/Ruminococcaceae/*Ruminococcus*	0.0080	-0.33

Table S3 | Different levels of taxa significantly (QIIME t-test, p<0.01) differentially abundant between microbial communities sampled from the 12vs. 12 extreme birds based on AME. High indicates that taxa are present only in high AME birds. Positive fold is more abundant in high AME birds.

**TRIAL 1**	GreenGenes taxonomy	p-value	FoldGood
denovo33326	Firmicutes/Bacilli/Lactobacillales/Lactobacillaceae/*Lactobacillus/Lactobacillus salivarius*	0.0062	Low
**TRIAL 1**	GreenGenes taxonomy	p-value	FoldGood
denovo32578	Firmicutes/Bacilli/Lactobacillales/Lactobacillaceae/*Lactobacillus/Lactobacillus reuteri*	0.0023	-5.66
denovo5661	Firmicutes/Clostridia/Clostridiales/Ruminococcaceae	0.0024	16.00
denovo14082	Firmicutes/Bacilli/Lactobacillales/Lactobacillaceae/*Lactobacillus/Lactobacillus reuteri*	0.0036	-8.00
denovo25651	Firmicutes/Clostridia/Clostridiales/Ruminococcaceae/*Faecalibacterium*	0.0047	3.22
denovo28886	Firmicutes/Bacilli/Lactobacillales/Lactobacillaceae/*Lactobacillus/Lactobacillus reuteri*	0.0069	-4.00
**TRIAL 2**	GreenGenes taxonomy	p-value	FoldGood
denovo28337	Firmicutes/Bacilli/Lactobacillales/Lactobacillaceae/*Lactobacillus/Lactobacillus reuteri*	0.0062	High
denovo7979	Firmicutes/Bacilli/Lactobacillales/Lactobacillaceae/*Lactobacillus/Lactobacillus salivarius*	0.0090	-9.00

Table S4 | Pearson correlations between different levels of taxa and AME values in all birds from each trail.

**TRIAL 1**	GreenGenes taxonomy	p-value	r
denovo29914	Tenericutes/Mollicutes/RF39	0.0021	-0.37
**TRIAL 2**	GreenGenes taxonomy	p-value	r
denovo28886	Firmicutes/Bacilli/Lactobacillales/Lactobacillaceae/*Lactobacillus/Lactobacillus reuteri*	0.0000	-0.50

denovo14082	Firmicutes/Bacilli/Lactobacillales/Lactobacillaceae/*Lactobacillus/Lactobacillus reuteri*	0.0033	-0.34
denovo32578	Firmicutes/Bacilli/Lactobacillales/Lactobacillaceae/*Lactobacillus/Lactobacillus reuteri*	0.0050	-0.33
denovo27306	Firmicutes/Bacilli/Lactobacillales/Lactobacillaceae/*Lactobacillus/Lactobacillus reuteri*	0.0092	-0.30
**TRIAL 3**	GreenGenes taxonomy	p-value	r
denovo23735	Firmicutes/Clostridia/Clostridiales/Ruminococcaceae	0.0049	-0.35
denovo15229	Firmicutes/Bacilli/Lactobacillales/Lactobacillaceae/*Lactobacillus/Lactobacillus crispatus*	0.0076	0.33
denovo8803	Firmicutes/Clostridia/Clostridiales/Lachnospiraceae/Coprococcus	0.0081	0.33

Table S5 | Different levels of taxa significantly (QIIME t-test, $p<0.01$) differentially abundant between microbial communities sampled from the 12vs. 12 extreme birds based on GR. High indicate that taxa are present only in high **GR** birds. Positive fold indicates higher abundance in high Growth Rate birds.

**TRIAL 1**	GreenGenes taxonomy	p-value	FoldGood
FAMILY	Firmicutes/Clostridia/Clostridiales/Ruminococcaceae	0.0082	2.37
GENUS	Firmicutes/Clostridia/Clostridiales/Ruminococcaceae/unknown	0.0093	2.26
denovo8806	Firmicutes/Clostridia/Clostridiales	0.0004	Low
denovo32512	Firmicutes/Bacilli/Lactobacillales/Lactobacillaceae/*Lactobacillus*	0.0023	Low
denovo10379	Firmicutes/Clostridia/Clostridiales/Ruminococcaceae	0.0094	-3.00
**TRIAL 2**	GreenGenes taxonomy	p-value	FoldGood
denovo4753	Firmicutes/Clostridia/Clostridiales/Ruminococcaceae	0.0115	Low

Table S6 | Pearson correlations between different levels of taxa and **GR** values in all birds from each trail.

**TRIAL 1**	GreenGenes taxonomy	p-value	r
FAMILY	Firmicutes/Clostridia/Clostridiales/Ruminococcaceae	0.0108	0.31
GENUS	Firmicutes/Clostridia/Clostridiales/Ruminococcaceae/unknown	0.0112	0.31
GENUS	Firmicutes/Clostridia/Clostridiales/Ruminococcaceae/*Faecalibacterium*	0.0299	0.27
denovo10379	Firmicutes/Clostridia/Clostridiales/Ruminococcaceae	1.47E-08	0.63
denovo37572	Firmicutes/Clostridia/Clostridiales/Ruminococcaceae/*Faecalibacterium*	0.0002	0.45
denovo10043	Bacteroidetes/Bacteroidia/Bacteroidales/Bacteroidaceae/*Bacteroides/Bacteroides fragilis*	0.0003	0.43
denovo32512	Firmicutes/Bacilli/Lactobacillales/Lactobacillaceae/*Lactobacillus*	0.0008	0.40
denovo8806	Firmicutes/Clostridia/Clostridiales	0.0011	0.39
denovo12953	Tenericutes/Mollicutes/RF39	0.0037	0.35
denovo8561	Firmicutes/Bacilli/Lactobacillales/Lactobacillaceae/*Lactobacillus*	0.0071	0.33
denovo6371	Tenericutes/Mollicutes/RF39	0.0092	0.32
**TRIAL 2**	GreenGenes taxonomy	p-value	r
GENUS	Firmicutes/Clostridia/Clostridiales/Lachnospiraceae/*Ruminococcus*	0.0020	-0.36
GENUS	Firmicutes/Clostridia/Clostridiales/Ruminococcaceae/*Clostridium*	0.0078	-0.31
denovo4753	Firmicutes/Clostridia/Clostridiales/Ruminococcaceae	0.0001	-0.44
denovo21177	Firmicutes/Bacilli/Lactobacillales/Lactobacillaceae/*Lactobacillus*	0.0046	-0.33
denovo12890	Firmicutes/Clostridia/Clostridiales/Lachnospiraceae/*Ruminococcus/Ruminococcus torques*	0.0054	-0.32
denovo38034	Firmicutes/Bacilli/Lactobacillales/Lactobacillaceae/*Lactobacillus*	0.0087	-0.30
**TRIAL 3**	GreenGenes taxonomy	p-value	r
denovo17576	Firmicutes/Clostridia/Clostridiales/	0.0033	0.36
denovo32969	Firmicutes/Bacilli/Lactobacillales/Lactobacillaceae/*Lactobacillus/Lactobacillus salivarius*	0.0052	0.35
denovo24223	Firmicutes/Bacilli/Lactobacillales/Lactobacillaceae/*Lactobacillus/Lactobacillus crispatus*	0.0071	0.33
denovo31632	Firmicutes/Bacilli/Lactobacillales/Lactobacillaceae*/Lactobacillus/Lactobacillus salivarius*	0.0072	0.33

denovo23888	Firmicutes/Bacilli/Lactobacillales/Lactobacillaceae/*Lactobacillus/Lactobacillus crispatus*	0.0079	0.33

Table S7 | Different levels of taxa significantly (QIIME t-test, p<0.01) differentially abundant between microbial communities sampled from the 12vs. 12 extreme birds based on FE. High indicates that taxa is present only in high **FE** birds. Positive fold indicates higher abundance in high FE, negative fold indicates higher abundance in low FE.

**TRIAL 1**	GreenGenes taxonomy	p-value	FoldHigh
denovo37369	Bacteroidetes/Bacteroidia/Bacteroidales/Bacteroidaceae/*Bacteroides/Bacteroides fragilis*	0.0076	-7.50
**TRIAL 2**	GreenGenes taxonomy	p-value	FoldHigh
FAMILY	Bacteroidetes/Bacteroidia/Bacteroidales/Bacteroidaceae	0.0053	-3.10
GENUS	Bacteroidetes/Bacteroidia/Bacteroidales/Bacteroidaceae/*Bacteroides*	0.0053	-3.10
denovo26333	Firmicutes/Bacilli/Lactobacillales/Lactobacillaceae/*Lactobacillus*	0.0100	High
denovo28895	Firmicutes/Clostridia/Clostridiales	0.0100	High
denovo27497	Firmicutes/Bacilli/Lactobacillales/Lactobacillaceae/*Lactobacillus/Lactobacillus reuteri*	0.0106	3.83
**TRIAL 3**	GreenGenes taxonomy	p-value	FoldHigh
denovo15167	Firmicutes/Clostridia/Clostridiales	0.003105	3.97
denovo27818	Firmicutes/Bacilli/Lactobacillales/Lactobacillaceae/*Lactobacillus/Lactobacillus crispatus*	0.006492	Low
denovo12985	Firmicutes/Bacilli/Lactobacillales/Lactobacillaceae/*Lactobacillus/Lactobacillus salivarius*	0.007045	8.71
denovo22961	Firmicutes/Clostridia/Clostridiales/Ruminococcaceae/*Faecalibacterium*	0.00931	-17.45

Table S8 | Pearson correlations between different levels of taxa and **FE** values in all birds from each trail.

**TRIAL 1**	GreenGenes taxonomy	p-value	r
FAMILY	Firmicutes/Bacilli/Lactobacillales/Lactobacillaceae	0.0062	0.33
GENUS	Firmicutes/Bacilli/Lactobacillales/Lactobacillaceae/*Lactobacillus*	0.0062	0.33
denovo37369	Bacteroidetes/Bacteroidia/Bacteroidales/Bacteroidaceae/*Bacteroides/Bacteroides fragilis*	0.0016	-0.38
denovo10043	Bacteroidetes/Bacteroidia/Bacteroidales/Bacteroidaceae/*Bacteroides/Bacteroides fragilis*	0.0016	-0.38
denovo11094	Firmicutes/Bacilli/Lactobacillales/Lactobacillaceae/*Lactobacillus/Lactobacillus salivarius*	0.0025	0.37
denovo11042	Firmicutes/Bacilli/Lactobacillales/Lactobacillaceae/*Lactobacillus/Lactobacillus salivarius*	0.0035	0.35
denovo33813	Firmicutes/Bacilli/Lactobacillales/Lactobacillaceae/*Lactobacillus*	0.0041	0.35
denovo38138	Firmicutes/Bacilli/Lactobacillales/Lactobacillaceae/*Lactobacillus/Lactobacillus salivarius*	0.0087	0.32
denovo33326	Firmicutes/Bacilli/Lactobacillales/Lactobacillaceae/*Lactobacillus/Lactobacillus salivarius*	0.0094	0.32
**TRIAL 2**	GreenGenes taxonomy	p-value	r
FAMILY	Bacteroidetes/Bacteroidia/Bacteroidales/Bacteroidaceae	0.0015	-0.36
GENUS	Bacteroidetes/Bacteroidia/Bacteroidales/Bacteroidaceae/*Bacteroides*	0.0015	-0.36
GENUS	Tenericutes/Erysipelotrichi/Erysipelotrichales/Erysipelotrichaceae/*Clostridium*	0.0032	-0.34
denovo4753	Firmicutes/Clostridia/Clostridiales/Ruminococcaceae	0.0005	-0.40
denovo7572	Firmicutes/Clostridia/Clostridiales/Ruminococcaceae	0.0007	-0.39
denovo15209	Bacteroidetes/Bacteroidia/Bacteroidales/Bacteroidaceae/*Bacteroides/Bacteroides fragilis*	0.0019	-0.36
denovo13545	Firmicutes/Clostridia/Clostridiales/Ruminococcaceae	0.0023	-0.35
denovo27999	Firmicutes/Bacilli/Lactobacillales/Lactobacillaceae/*Lactobacillus/Lactobacillus crispatus*	0.0077	0.31
denovo27497	Firmicutes/Bacilli/Lactobacillales/Lactobacillaceae/*Lactobacillus/Lactobacillus reuteri*	0.0094	0.30
**TRIAL 3**	GreenGenes taxonomy	p-value	r
denovo7211	Firmicutes/Bacilli/Lactobacillales/Lactobacillaceae/*Lactobacillus/Lactobacillus crispatus*	0.0048	-0.35
denovo32968	Tenericutes/Mollicutes/RF39	0.0057	-0.34

References

Huson, D.H., Auch, A.F., Qi, J., and Schuster, S.C. (2007). MEGAN analysis of metagenomic data. *Genome Res* 17**,** 377-386.

Stanley, D., Geier, M.S., Hughes, R.J., Denman, S.E., and Moore, R.J. (2013). Highly variable microbiota development in the chicken gastrointestinal tract. *PLoS One* 8**,** e84290.
